# Oxidative Stress and Total Phenolics Concentration in COPD Patients—The Effect of Exercises: A Randomized Controlled Trial

**DOI:** 10.3390/nu14091947

**Published:** 2022-05-06

**Authors:** Katarzyna Domaszewska, Sara Górna, Malwina Pietrzak, Tomasz Podgórski

**Affiliations:** Department of Physiology and Biochemistry, Poznan University of Physical Education, 61-871 Poznan, Poland; gorna@awf.poznan.pl (S.G.); m.pietrzak@awf.poznan.pl (M.P.); podgorski@awf.poznan.pl (T.P.)

**Keywords:** COPD, TBARS, allantoin, total phenolics

## Abstract

Patients with chronic obstructive pulmonary disease (COPD) suffer from exercise intolerance, the sensation of dyspnea, and fatigue, which are the main reasons for limiting their physical activity. In addition to changes in the respiratory and circulatory systems in patients with COPD, peripheral muscle dysfunction, with numerous metabolic dysfunctions, is observed. One of the symptoms of the described anomalies, among others, is an antioxidative and prooxidative imbalance. The aim of the study was to demonstrate the impact of endurance training, carried out in the extended pulmonary rehabilitation program in COPD patients, on the imbalance between prooxidants and antioxidants in their bodies. Methods: The tests were carried out on a group of patients (*n* = 32) with COPD; 20 randomly selected people underwent a modified rehabilitation program during their rehabilitation stay, and the obtained results were compared with the results of 12 patients (control group) who were treated without endurance training. At the beginning and at the end of the study, spirometry and cardiopulmonary exercise tests (CPET) were performed. Oxidative stress (allantoin (All) and substances which react with thiobarbituric acid) and antioxidant (ferric reducing ability of plasma and total phenolics) parameters’ concentrations were determined in the venous blood. Results: In the study group, greater post-training increases of VO_2max_ (*p* = 0.0702) and FEV_1_/FVC (*p* < 0.05; ES: 0.436) were reported. The applied CPET at each time caused an increase in the All concentration (*p* < 0.05) in the study and control groups. Conclusions: Endurance training applied as a part of the rehabilitation process did not cause the additional aggravation of oxidative stress and blood total phenolics concentration.

## 1. Introduction

The participation of hypoxemia in the severity of inflammatory processes occurring within the respiratory tract in patients with chronic obstructive pulmonary disease (COPD) is well-known and described in the literature [1]. Free radicals are the initiators of the production of proinflammatory markers in the body, while inflammatory cytokines stimulate xanthine oxidase (EC 1.17.3.2) and NADPH oxidase (EC 1.6.3.1), indirectly reducing the antioxidant capacity of plasma [2]. Thus, it has been shown that the decrease in the ferric reducing ability of plasma (FRAP) concentration occurs during exacerbations of the disease [3]. Changes caused by the increased production of free radicals may be reversible. The microscopic image of the damaged tissue showed slight swelling, hypertrophy of the endoplasmic reticulum, mitochondria shrinkage, damage of ribosomes, and the aggregation of chromatin around the nucleus. However, when the rate of production of free radicals is higher than the rate of recovery mechanisms, a significant shrinking of mitochondria may occur, with comb mitochondrial damage, dispersal of cellular elements, and cytoplasmic membrane damage. Extensive cellular changes lead to their necrosis [4]. The source of free radicals can be mitochondrial reactions, the xanthine system, or disintegrating granulocytes. Some free radicals, as well as harmful products of lipid oxidation (e.g., malondialdehyde (MDA)), affect signal transduction mechanisms and active gene expression of inflammatory markers, thereby exacerbating the severity of the inflammation and disease processes. For this reason, the problem of the antioxidants’ therapeutic role in preventing the effects of reactive oxygen species (ROS) on cells and tissues are extensively discussed. Gosker et al. [5] observed a 26% increase in the level of antioxidants on average, including vitamin E, glutathione, uric acid, and TBARS, in COPD patients in comparison with healthy persons. In the foregoing studies, the change in concentration of MDA in the blood of these patients was not demonstrated, which was explained by the adaptation of the cells to function in an environment with an increased amount of free radicals. This hypothesis is somewhat different from that presented earlier by Rahman et al. [6]. It has been shown in many publications that a higher concentration of total phenolics in the blood protects the body against many civilization diseases (e.g., atherosclerosis and cancer) [7,8,9]. The results of tests carried out on the French population are particularly significant, as the French are renowned for their habit of drinking red wine during the consumption of meals, containing a large amount of total phenolics. Despite a high-fat diet, French people are significantly less likely to die from cardiovascular problems than representatives of other European countries (the so-called French Paradox). It should be noted that phenolic compounds are only derived from plant sources (e.g., olive oil, green and black tea, red wine, and cranberry) [10]. Currently, there are over 8000 substances of this type known to have strong antioxidant properties [11].

One of the most sensitive markers of the severity of free radicals occurring in the body is allantoin. It is the product of non-enzymatic oxidation of uric acid, resulting from the degradation of purine nucleotides [12]. Allantoin may be excreted in the urine or alternately converted in the cycle into urea and as such eliminated from the body [13]. Allantoin, being a free radical, damages cell membranes in the body and, together with substances which react with thiobarbituric acid (TBARS), is considered an indicator of the severity of free radical processes in the body. Mikami et al. observed the increase in the concentration of allantoin 30 min after exercising at 100% intensity in the absence of VO_2_max, with no changes in the concentration of TBARS, and concluded that allantoin is a better indicator of free radical changes than TBARS [13].

Physical training is widely recognized as the basis of rehabilitation programs improving skeletal muscles in patients with COPD and other pulmonary diseases. A properly conducted rehabilitation process is completely safe for the patient, but one should take into account the possibility of adverse symptoms and exacerbation of the disease. This is often due to the lack of individualization of the training program and the use of too high intensity efforts. In order to maintain safety and to reduce the occurrence of adverse events, it is recommended to perform a cardiac exercise test. It enables the determination of safe maximal exercise loads and shows the individual physiological response of the tested person to the application of the test load. Additionally, during each training session, patients should be monitored for their cardiological parameters, blood oxygen saturation, intensity of dyspnea, and subjective feeling of fatigue [14,15].

Positive effects in the form of an increase in physical capacity are brought by training on the edge of the patient’s anaerobic changes threshold. In COPD patients, it may be difficult to achieve 30 min exercise times or desired intensity levels, even under close supervision by a specialist. An alternative in this case is interval training. Many rehabilitation programs are limited to training the lower limbs only. The inclusion in the training program of exercises involving the muscles of the upper limbs avoids the feeling of dyspnea and limits the increase in minute ventilation of the lungs during the lifting of the arms.

The use of training sessions, including resistance or strength exercises, is beneficial in terms of improving muscle potential and strength. The intensity of such exercises should not exceed 50–85% of the maximal voluntary contraction (MVC). Such a training program is often better tolerated by the patient than traditional endurance training. The combination of both types of training seems to be the most beneficial and effective method of rehabilitation of COPD patients as it leads to an improvement in muscle strength and endurance [16]. It is clear that low-intensity training programs improve the patient’s daily activities, but the physiological effect is only visible with higher-intensity training programs. The use of lower-intensity efforts takes into account the presence of disease symptoms in patients that limits their exercise capacity, and at the same time causes a stronger motivation for the long-term use of these rehabilitation programs. It has been found experimentally that to induce post-training physiological effects in patients with COPD, it is sufficient to exercise at the level of 60% VO_2_ peak [14,15,16].

The standard treatment of COPD patients includes the administration of oxygen at rest and during training. Hanneke et al. [17], in his elaboration on the effects of oxygen on the antioxidant potential of plasma and the inflammatory marker concentrations in the blood, stated that the administration of oxygen to COPD patients during exercise reduces the severity of oxidative stress and the development of inflammatory changes. It can be said, on the basis of these results, that supporting the patient by the administration of oxygen increases the positive effects of rehabilitation. At the same time, the things that tend to decrease it include the frequency of exacerbations and the number of hospitalizations in this patient group. The results of tests conducted by Carpaganano et al. and Phillips et al. provided different findings [18,19]. They showed that supplementation with pure oxygen caused an increase in the concentrations of interleukin (IL)-6 and isoprostans, as well as the severity of antioxidant stress, both in COPD patients and in healthy people. The aim of the study was to demonstrate the impact of endurance training, carried out in the extended pulmonary rehabilitation program of COPD patients, on the imbalance between blood concentrations of prooxidative and antioxidative markers.

## 2. Materials and Methods

### 2.1. Participants

This study was a controlled randomized trial. Randomization was performed as simple random allocation: each subject identifier was forwarded to a person who was not involved with the conduct of the study and who performed the randomization blindly using a computer list.

**Figure 1:**
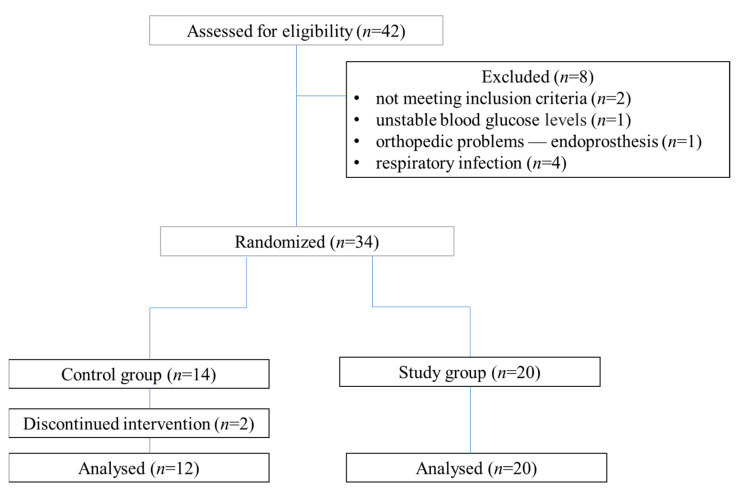
Patient flow chart.

The study was conducted according to the Declaration of Helsinki and the National Statement and Human Research Ethics Guidelines and approved by the Institute for Research in Biomedicine (IRB) at the Poznan University of Medical Sciences (3 February 2005; Ethics Approval Number: 236/05). The exercise stress test laboratory was adequately equipped to provide advanced life support in the event of a cardiac arrest. Patients were referred by their respiratory physician practitioners. Inclusion criteria for the study were pulmonary insufficiency with COPD and qualification for lung rehabilitation. It is beneficial to start the rehabilitation program as soon as the flare-up of the disease is under control, preferably in health-care settings, including hospitals. Eligibility for the rehabilitation program and the method of its implementation also depended on the course of the disease, the pharmacological treatment, and the possibility of cooperation with the patient. Exclusion criteria were the presence of advanced chronic complications of diabetes; severe cardiovascular or orthopaedic disease; active or post-cancerous disease (ongoing radiation/chemotherapy treatment); liver diseases (ALT > 3× upper limit of normal), except for patients with fatty liver disease; or psychological disorders. After completing the baseline screening and evaluation, eligible patients were randomly assigned to a proper group. An investigator not involved in subject recruitment developed a computer-generated random allocation schedule and placed the assignments in sealed, sequentially numbered envelopes. As each patient entered the trial, the next envelope in the sequence was opened, indicating which treatment the patient would undergo. The patient flow chart is shown in Figure 1.

**Figure 2:**
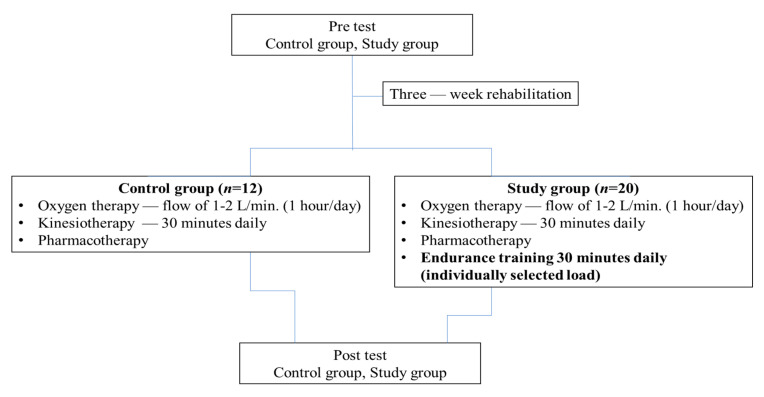
Research protocol.

The first group of patients (study group, *n* = 20) during the 3-week long rehabilitation stay, in addition to standard oxygen therapy, kinesiotherapy, and pharmacotherapy, underwent daily 30 min long endurance training with individually selected loads that were appropriate to the patient’s health status. The pharmacological treatment of COPD relies mostly on symptomatic treatment, i.e., inhaled anticholinergic agents, β2-agonists, and phosphodiesterase inhibitors, aiming at their bronchodilatation capacity. Inhaled corticosteroid therapy is mainly prescribed in far advanced stages of the disease. The current pharmacological treatment was not modified in the patients during the experiment. The heart rate while exercising on a cycle ergometer in the study group was 50–70% of the predicted HRmax, taking into account the patient’s age. The results were compared with the results of 12 randomized patients (control group), who were treated only using oxygen therapy, kinesiotherapy, and pharmacotherapy, without endurance training (Figure 2).

All the participants were asked to carefully read and sign an informed consent form, taking precautions to protect their privacy. All of the patients from the sample group gave their written informed consent.

### 2.2. Pulmonary Function Tests

The evaluation of pulmonary function was performed by conventional spirometry using a spirometer (abc Spec PC, RS 2000 device by abcMED Corporation, Cracow, Poland). The directly evaluated parameters were lung flows; forced vital capacity (FVC), performed three times, according to the standards of the American Thoracic Society (ATS) and the European Respiratory Society (ERS) in the sitting position [20]. Results were expressed as absolute values and as percentages of the reference predicted values from Pereira et al. [21]. The FVC procedure allowed for the determination of the forced expiratory volume in 1 s (FEV_1_) and FEV_1_/FVC ratio. Examinations were conducted two times in the course of the project (at the beginning and at the end of the treatment).

### 2.3. Methodology of Marking Exercise Tolerance of Patients

The exercise tests were conducted between 08:00 h and 12:00 h in an air-conditioned laboratory 2 h after consuming a light breakfast (one sandwich with butter and cheese; approximately 200 kcal). Each of the studied participants performed an exercise with increasing intensity. The output load for each person included walking at a speed of 3 km/h on a treadmill (Woodway ES1; Woodway, Waukesha, WI, USA). Then, each minute the intensity of the walk was increased by 1 km/h, which was continued until exhaustion. Progressive incremental exercise testing was discontinued when the subjects displayed marked breathlessness, or ECG changes showing ST segment depression greater than 2 mm or a short run of premature ventricular contractions. During the exercise test, cardiorespiratory parameters were continuously recorded. Heart rate (HR) was recorded using a sport-tester by Polar Accurex Plus device (Polar Elektro, Tampere, Finland). The minute ventilation (VE) of the lungs and oxygen consumption per minute (VO_2_) were measured using a portable gas analyzer (German Jeager Oxycon Mobile, Viasys Healthcare, Höchberg, Germany). In addition, during the exercise test distance, the duration of exercise and speed of walking were measured and recorded. On the basis of the exercise-induced oxygen consumption and heart rate values, individual values of VO_2_max were calculated [22]. The person executing the CPET had certificates and authorizations to perform the tests.

### 2.4. Preparation of Blood Samples for Analysis

In each of the test dates, venous blood was taken twice from the ulnar veins i.e., at rest (fasting blood; 07:00 h and 5 min after finishing the exercise using a S-Monovette syringe (Sarstedt, Nümbrecht, Germany)). The blood was centrifuged at 1500 g nt 4 °C for 5 min (Universal 320R; Hettich Lab Technology, Tuttlingen, Germany), and the collected plasma samples were frozen and stored at −80 °C until the analysis (U410, Ultra-Low Temperature Freezer, New Brunswick Scientific, United States). All reagents used for the measurements of the above parameters were obtained from Sigma-Aldrich (Chemie GmbH, Steinheim, Germany).

#### 2.4.1. Determination of Allantoin

Marking was made using the HPLC (high-performance liquid chromatography) method with a lamp at a wavelength of λ = 360 nm. A total of 250 μL plasma was neutralized in 125 μL 1.6 mmol/L HClO_4_ (POCH, Gliwice, Poland). Then, the sample was centrifuged, and 250 μL of the resulting supernatant was transferred to a glass tube. Then, 250 μL 0.12 M NaOH (POCH, Gliwice, Poland) was added. The sample was boiled for 20 min at 100 °C (water bath, JWE-357, Warsaw, Poland). Then, 250 μL 1 M HCl (POCH, Gliwice, Poland) and 50 μL DNP (2,4-dinitrophenol, POCH, Gliwice, Poland) were added, and the sample was boiled again for 5 min at 100 °C. The carrier phase was determined by a buffer with pH = 4.75 and composition of 55% sodium citrate (POCH, Gliwice, Poland) and sodium acetate (POCH, Gliwice, Poland), as well as 45% methanol (POCH, Gliwice, Poland). The flow rate was 1.0 mL/min. A quantitative analysis was performed similarly as in the case of marking ox purines. The resting allantoin concentration in plasma was 3.1–36.4 μmol/L [23]. A Hewlett-Packard 1050 apparatus with UV detector (Ramsey, NJ, USA) was used.

#### 2.4.2. Determination of FRAP

A method developed by Benzie et al. [24] was used in the study. It involves a reduction of the Fe^3+^TPTZ (2,4,6-tripyridyl-s-triazine; Sigma-Aldrich, Gillingham, UK) complex to a blue complex of Fe^2^+-TPTZ. The color intensity of the resulting solution is directly proportional to the antioxidant power of plasma. A total of 10 μL plasma was diluted with 30 μL deionized water, followed by adding 300 μL reactive solution (2,4,6-tripirydylo-s-triazine (TPTZ) + ferric chloride III (FeCl_3_·6H_2_O; POCH, Gliwice, Poland) + acetate buffer (pH = 3.6, reagents from POCH, Gliwice, Poland)). After 6 min of incubation at 37 °C, the absorbance was determined on a multi-detector microplate ELISA reader (Synergy 2 SIAFRT, BioTek, Winooski, VT, USA) at λ = 593 nm. The standard curve was established using a stoichiometrically diluted solution of iron sulphate II (FeSO_4_·7H_2_O; POCH, Gliwice, Poland). The total antioxidant capacity of plasma (FRAP) had the following reference values at rest: 600–1600 μmol/L.

#### 2.4.3. Determination of TBARS

The method developed by Ohkawa et al. [25] was used in the marking. This method involves the condensation of MDA with thiobarbituric acid, and the formation of a dye compound with 50 μL plasma, 50 μL sodium dodecyl sulphate (SDS; POCH, Gliwice, Poland), 375 mL 20% acetic acid (POCH, Gliwice, Poland), and 375 mL 0.8% thiobarbituric acid (Sigma-Aldrich, Gillingham, UK) was placed in a water bath at 95 °C for 60 min. After incubation, the sample was cooled, and the elution was made into a solution of a dye compound of n-butane (POCH, Gliwice, Poland). After centrifugation, the upper layer of the solution was separated, and the measurement was performed on a multi-detector microplate ELISA reader (Synergy 2 SIAFRT, BioTek, Winooski, VT, USA) at λ = 532 nm. The standard curve was created from a stoichiometrically diluted solution (1,1,3,3-tetramethoxypropane (TMP; Sigma-Aldrich, St. Louis, MI, USA). The concentration of TBARS in the plasma of healthy people was 1–6 μmol/L.

#### 2.4.4. Determination of Total Phenolics

The concentration of phenolic compounds in the blood was determined using the method developed by Singleton and Rossi [26]. This method exploits the ability of oxidation of phenolic groups by the Folin-Ciocalteu reagent (Chemour, Piekary Śląskie, Poland). The resulting compounds are converted to a blue complex. The color of the solution was measured using a Marcel Media plus spectrophotometer (Marcel sp. z o.o., Zielonka, Poland) at λ = 765 nm. The standard curve was established using standard solutions of gallic acid (GAE; Sigma-Aldrich, St. Louis, MI, USA). The concentration of total phenolics was expressed as a GAE equivalent in g/L of plasma. The proper resting reference values were assumed to be the concentration of total phenolics, which was equal to 2.8–4.0 g GAE/L.

### 2.5. Statistical Analysis

The sample size was calculated based on data from the study of Najafi Mehri S et al. After calculation of the power analysis of the Mann–Whitney test (adopting a power as 1-beta error probability: 80%, effect size: 1.79, and error assumed as alpha: 0.05 (two-sided), 6 participants were indicated for allocation equally for each test. We decided to accrue more participants in each test, due to possible dropout [27]. The data are presented as means and standard deviations (SD). The obtained results were analyzed statistically using the Dell Statistica data analysis software system (version 13, Dell Inc., Round Rock, TX, USA). The normality of distributions was verified using the Shapiro–Wilk test. The Mann–Whitney U test and the Wilcoxon test were employed for non-normally distributed variables, respectively, to evaluate the significance of differences between the groups and test dates. Spearman’s rank analysis was used to calculate correlation coefficients. The level of statistical significance was set at *p* ≤ 0.05. Effect sizes [ES] were calculated as the difference between means divided by the pooled standard deviation. Using Cohen’s (1988) criteria, an ES ≥ 0.20 and <0.50 was considered small, ≥0.50 and <0.80 medium, and ≥0.80 large [28].

## 3. Results

Finally, the results of 32 patients (16 male, 16 female) were subjected to a statistical analysis. The detailed anthropometric characteristics of the respondents are shown in Table 1. The study groups did not differ significantly in terms of the anthropometric indicators tested in the first period of the study. COPD was classified as moderate to severe.

During the test, patients stayed at the Wielkopolska Centre of Pulmonology and Thoracosurgery in Poznan, Poland. Therefore, the impact of diet on the potential plasma antioxidant values can be excluded in the study. During the experiment, the patients consumed the same meals prepared and delivered by the catering company. The subjects were also asked not to consume additional food rations and dietary supplements for the duration of the project and not to perform any additional physical activity. The study group underwent the same series of spirometry and exercise twice, at the beginning and at the end of the rehabilitation stay (3 weeks). The results are given in Table 2. It presents, on the basis of average values, the post-training changes of exercise parameters examined in the study and control groups. While analyzing the results, it is visible that the use of additional endurance training in patients positively affected the distance covered by them in the exercise test, the value of VO_2_max, and the lung spirometry parameters.

The nature of changes in the resting values of selected biochemical markers and the differentiated response to the exercise test between the study and control groups on two dates of studies are shown in Table 3.

In the first research period (at the beginning of the treatment), a high correlation was demonstrated for the entire study group (*n* = 32) between the VO_2_max value and the distance covered in the exercise test (r= 0.7517, *p* < 0.001) and walking speed (r = 0.7732, *p* < 0.001). Moreover, a correlation between the resting concentration of total phenolics and VO_2_max (r = −0.4386, *p* < 0.05), total phenolics and FRAP concentration (r = 0.3915, *p* < 0.05), and total phenolics and TBARS concentration (r = 0.4302, *p* < 0.05) was also demonstrated.

Additionally, the endurance training used in the study group did not cause significant changes in the results of exercise tests and the concentrations of the tested biochemical indices in the blood. Only a significant change in FEV_1_/FVC values was recorded (*p* = 0.0011) (Table 2 and Table 3). The post-training change (∆ VO_2_max) correlated with the change in distance (∆ distance) (r = 0.5338, *p* < 0.05) and walking speed (∆ velocity) (r = 0.5850, *p* < 0.05), as well as the change in resting FRAP concentration (∆) (r = −0.5751, *p* < 0.05).

In the control group, there was a significant decrease in the distance covered by the patients (*p* < 0.05). The difference in the distance covered between the study dates correlated with the change (∆) of the resting blood total phenolics concentration in this group of patients (r = 0.7783, *p* < 0.05). The changes in the FEV_1_/FVC index between the study dates correlated with the increase in the concentration of blood allantoin during exercise (r = 0.6833, *p* < 0.05).

The analysis of changes between the groups showed only a significant difference in the distance covered (*p* = 0.0102; ES: 0.0960) and walking speed in the exercise test (*p* = 0.028; ES: 0.9750) (Table 2.)

## 4. Discussion

On the basis of our own studies of patients with moderate levels of COPD, one found very low FRAP concentrations, equal to 693.08 ± 158.15 μmol/L and equal to 917.88 ± 165.72 μmol/L for the control group, respectively [29]. In addition, in our previous studies on a group of patients with COPD, one found very low FRAP concentrations, equal to 567 ± 131 μmol/L at an FEV_1_/FVC ratio equal to 40% [30]. In their extensive studies on oxidative stress in COPD, Nadeem et al. [31] found lower concentrations of FRAP and the activities of both glutathione peroxidase (EC 1.11.1.9) and superoxide dismutase (EC 1.15.1.1), with a simultaneous increase in the concentration of the superoxide anion (O_2_∙-) in plasma and glutathione, compared to healthy persons. In addition, they showed a statistically significant negative correlation between the concentration of FRAP and the FEV_1_/FVC ratio. Physical activity, not only in this group of patients but also in healthy people who perform intense physical effort, caused a 20-fold increase in oxygen consumption compared to resting conditions. Mitochondrial reactions were accelerated, resulting in the increase in ROS production. Excessive production of reactive forms of mitochondrial oxygen, as well as the products of the consumption of antioxidants, and the change of FRAP concentrations of reactions catalyzed by xanthine oxidase, led to an unsustainable prooxidative and antioxidant balance. The test effort to which the patients were subjected resulted in a statistically insignificant increase in the concentration of FRAP in the study group, both in the first period and in the second period of the study. In contrast, the increase in FRAP concentrations in the second period of study in the control group proved to be statistically significant (*p* = 0.027). An exercise-induced increase in the activities of antioxidant enzymes is not due to the severity of their de novo synthesis, but is caused by the lack of inhibitors or the presence of activators modifying the rate of enzymatic reactions [32]. This may indicate a stronger stress disorder of prooxidative and antioxidant balance in the group that did not undergo endurance training. The literature on the impact of one-off exercise and training on plasma antioxidant enzymes is broad, and the data shown are controversial and often contradictory. Hence, there is difficulty in drawing clear conclusions in the practical application of the FRAP concentration analysis in order to assess the effectiveness of training or the body’s response to exercise [33]. Studies carried out by Miyazaki et al. and Woźniak showed that both high-speed training and endurance training caused an increase in the plasma antioxidant potential in healthy persons [32,33]. As a result of using endurance training in conjunction with oxygen therapy in the case of patients with COPD, one did not find any change in the concentration of FRAP in the study group, while the standard rehabilitation program, with the exception of aerobic exercise, caused a decrease in the concentration of plasma antioxidant potential from 917.89 ± 165.72 μmol/L to 761.86 ± 175.82 μmol/L (*p* = 0.099). It can be assumed that oxygen therapy results in a greater consumption of antioxidants, and the physical training used in COPD patients reduces this negative process. Another method evaluating cell oxidative stress is to measure the concentration of allantoin in the blood. In the available literature, there are no data on the effect of pulmonary rehabilitation on allantoin concentration in the blood. Hence, it is difficult to refer to the results of other researchers. In our previous studies on patients with severe COPD, one found a very high concentration of allantoin in the blood, equal to 40.07 ± 23.68 μmol/L, which was in significant excess of the reference value standards for this parameter. Concentrations of allantoin observed in these studies were up to three times higher than average concentrations of this parameter in the blood of healthy persons [34]. Similar concentrations of this metabolite were also observed in the present studies, and they were 46.31 ± 29.15 μmol/L for the study group, with an average FRAP concentration equal to 698.08 ± 162.26 μmol/L, and 24.90 ± 18.63 μmol/L for the control group, with an average FRAP concentration equal to 917.89 ± 165.73 μmol/L, respectively.

The results clearly show the resting disorder of the antioxidant potential of the body in patients with average COPD. According to the studies, none of the models of rehabilitation beneficially affected the prooxidative and antioxidant conditions of the organism. Similar to the analysis of blood FRAP concentration, there were no statistically significant changes in the resting allantoin concentrations or differences in exercise-induced increases in this parameter in both groups, as well as in both periods of study. However, in both groups and on each of the study terms, the test effort to which patients were subjected caused a statistically significant change in the allantoin concentration in the blood (*p* < 0.05), without a significant change in the concentration of TBARS. A similar regularity was described by Mikami et al. in their elaboration. They believed that allantoin is a better indicator of free radical changes taking place in the cells than the analysis of TBARS concentrations. During their studies on a group of healthy people, they observed an increase in the concentration of allantoin after 30 min of training with an intensity of 100% VO_2_max, with no change in the concentration of TBARS [13]. However, despite the lack of statistically significant differences in the change in resting concentrations of allantoin between the first and the second periods of the study in both groups, one demonstrated a statistically significant correlation (r = 0.7167, *p* < 0.05) between the change in the exercise-induced allantoin increase in the blood of the studied patients and the change in the VO_2_max after 3 weeks of rehabilitation. The study also showed a significant correlation between the change of exercise-induced allantoin and FRAP concentration for the study group (r = 0.6833, *p* < 0.05). It can, therefore, be concluded that the decrease in the level of exercise tolerance has a strong effect on the concentration of free radicals in the body, and they are also the main cause of pathological changes at the cellular level. Therapeutic increases of the VO_2_max in patients with COPD result in a decrease in oxidative stress, which can also be assessed indirectly in plasma lipoproteins and biological membranes.

The TBARS concentration depends not only on the speed of its production but also on the rate of its utilization in the liver. In the studied group of patients, the resting TBARS concentration fell within the physiological standards, while the post-training and post-rehabilitation changes were not statistically significant. This may indicate a lack of integrity of cell membranes, as a result of both the applied oxygen therapy and endurance training. An ability of the body to defend against harmful free radicals depends largely on the amount of antioxidant vitamins (A, C, and β-carotene), total phenolics, and trace elements (selenium, zinc, and manganese) in the blood [35,36]. Exercise training and increased oxidative stress occurring in COPD patients increase the organism’s need for these components. Although it is a known fact that increased amounts of antioxidants in the diet reduce the symptoms of oxidative stress, there is no guidance in the literature referring to the accurate amount needed. It probably depends on the severity of the type of illness and the intensity of physical activity undertaken by the patient [37]. Our own studies show that COPD patients have a two-fold lower resting blood concentration of total phenolics when compared with healthy persons. Only in the case of the analysis of exercise-induced changes in the concentration of total phenolics in the blood of the studied group, one found a statistical significance in the first period of the study (*p* = 0.009). In contrast, decreases in the resting concentration of this parameter after a 3-week rehabilitation period were not statistically significant in both groups. It can, therefore, be assumed that the applied rehabilitation programs did not significantly affect the resting concentration of total phenolics in the blood. Thus, in order to improve the antioxidant capacity of plasma, it would seem advantageous to incorporate food-source polyphenols or phenolics into the rehabilitation program. It has been shown in numerous studies that the administration of antioxidants increases the antioxidant potential of plasma [38,39].

## 5. Conclusions

Intense oxidative stress was observed in the group of patients with COPD, as measured by means of blood TBARS and allantoin concentrations. The concentration of allantoin was a more sensitive marker of the aggravation of free radical changes within the cell. Endurance training applied as a part of the rehabilitation process did not cause additional aggravation of the oxidative stress. From a prevention and protection standpoint, the application of supplementation and/or enriching the diet with antioxidative compounds in COPD patients to a varied degree of severity seems to be favorable.

### Limitations of the Study

Our study has some potential limitations. First, our study design lacked a sham or non-intervention control group. Second, the treatment intensity and sessions were based on previous relevant studies. We do not know if a greater number of sessions or treatment intensity would have revealed greater changes in outcomes or differences between the two groups.

## Figures and Tables

**Table 1:** Participants’ anthropometric characteristics (mean (SD)).

	Study Group (*n* = 20)	Control Group (*n* = 12)	*p*-Value
Age(years)	62.10 (11.28)	60.33 (9.61)	0.6262
Body weight (kg)	79.40 (19.39)	79.50 (11.98)	0.8761
Body height (cm)	166.45 (8.10)	169.92 (7.01)	0.2490
BMI (kg/m^2^)	28.39 (5.98)	27.38 (2.00)	0.4478

SD: standard deviations; BMI: Body Mass Index.

**Table 2:** The effect of 30 min daily endurance training on spirometric parameters (FEV_1_/FVC), VO_2_max, and distance covered (mean (SD)) on the treadmill test.

	Study Group			Control Group		
Variable	I	II	*p*-Value	I	II	*p*-Value
FEV_1_/FVC (%)	60.52 (16.39)	67.43 (15.31)	0.0010 (ES: 0.436)	54.05 (15.99)	58.02 (19.12)	0.3017
Distance (km)	0.28 (0.12)	0.33 (0.11)	0.1141	0.24 (0.10)	0.19 (0.13)	0.0179 (ES: 0.431)
Velocity (km/h)	4.33 (0.79)	4.66 (0.69)	0.0801	3.96 (0.80)	3.82 (0.96)	0.1088
VO_2_max (mL/kg/min)	19.77 (5.74)	21.37 (5.81)	0.0702	15.38 (4.76)	15.32 (5.02)	0.8240

I: first research period; II: second research period; ES: effect sizes; SD: standard deviations; VO_2_max: maximal oxygen uptake; FEV_1_/FVC: Tiffeneau–Pinelli index, describing correspondence between the forced expiratory volume.

**Table 3:** The effect of exercise tests and 30 min daily endurance training on plasma allantoin, FRAP, TBARS, and total phenolics concentration (mean (SD)) in patients with COPD.

		Study Group			Control Group		
Variable	Period	Rest	Post-Exercise	*p*-Value	Rest	Post-Exercise	*p*-Value
Allantoin (μmol/L)	I	46.31 (29.15)	63.88 (29.50)	0.0030 (ES: 0.599)	24.90 (18.63)	46.06 (27.97)	0.0022 (ES: 0.890)
	II	40.69 (27.73)	54.14 (25.73)	0.0031 (ES: 0.503)	26.37 (18.10)	43.87 (22.60)	0.0022 (ES: 0.855)
FRAP (μmol/L)	I	693.08 (158.15)	718.65 (134.26)	0.6149	917.88 (165.72)	953.75 (141.07)	0.3269
	II	689.95 (146.83)	706.44 (135.12)	0.8228	761.86 (175.82)	781.29 (173.57)	0.0277 (ES: 0.111)
TBARS (μmol/L)	I	3.10 (20.97)	3.07 (1.05)	0.2477	4.65 (1.23)	4.80 (1.34)	0.2361
	II	3.55 (1.57)	3.29 (1.25)	0.1729	5.96 (1.52)	5.51 (0.91)	0.4468
Total phenolics (g GAE/L)	I	1.91 (0.19)	2.11 (0.22)	0.0098 (ES: 0.973)	2.73 (0.40)	2.69 (0.54)	0.8589
	II	1.83 (0.39)	1.99 (0.39)	0.0526	2.69 (0.42)	2.77 (0.38)	0.3105

I: first research period; II: second research period; ES: effect sizes; FRAP: ferric reducing ability of plasma; TBARS: thiobarbituric acid reactive substances.

## Data Availability

The data presented in this study are available on request from the corresponding author. The data are not publicly available due to the consent provided by participants on the use of confidential data.
